# Creation and validation of a new tool for the monitoring efficacy of neurogenic bowel dysfunction treatment on response: the MENTOR tool

**DOI:** 10.1038/s41393-020-0424-8

**Published:** 2020-01-27

**Authors:** Anton Emmanuel, Klaus Krogh, Steven Kirshblum, Peter Christensen, Michele Spinelli, Dirk van Kuppevelt, Rainer Abel, Dietrich Leder, Bruno Gallo Santacruz, Kimberly Bain, Valentina Passananti

**Affiliations:** 1 GI Physiology Unit, University College Hospital, London, UK; 2 Department of Hepatology and Gastroenterology, Aarhus University Hospital, Aarhus, Denmark; 3 Kessler Institute for Rehabilitation, Rutgers New Jersey Medical School, New Jersey, USA; 4 Pelvic Floor Unit, Aarhus University Hospital, Aarhus, Denmark; 5 Spinal Unit, Niguarda Hospital, Milan, Italy; 6 Sint Maartensclinic Rehabilitation Center, Nijmegen, Netherlands; 7 Department of Orthopedic Surgery, Kinikum Bayreuth, Bayreuth, Germany; 8 Department Of Proctology and Endoscopy, Viszera Chirugie Zentrum, Munich, Germany; 9 Department of Clinical Development, Coloplast A/S, Humlebæk, Denmark; 10 Certified Facilitator, BaingGroup Consulting, Ontario, Canada

**Keywords:** Spinal cord diseases, Spinal cord diseases, Therapeutics

## Abstract

**Study design:**

Prospective observational study.

**Objectives:**

A tool to help decision-making tool for Neurogenic Bowel Dysfunction (NBD) in individuals with SCI is needed. We present a project to create and validate a new tool, the Monitoring Efficacy of NBD Treatment On Response (MENTOR), and to determine its level of concordance with decisions made by experienced clinicians in the field.

**Setting:**

UK, Denmark, USA, Italy, The Netherlands, Germany.

**Methods:**

The first phase was creation of the tool through a modified Delphi process. The second phase was the validation, wherein individuals with spinal cord injury with NBD were asked to complete the MENTOR tool immediately prior to clinic consultation. From the responses to the questionnaire of the tool, each participant was allocated into one of three categories reflecting the possible therapeutic recommendations (“recommend change”, “further discussion” and “monitoring”). An expert clinician then assessed the participant, blinded to MENTOR results, and made an independent treatment decision.

**Results:**

A total of 248 MENTOR forms were completed. Strong agreement was found when the MENTOR tool recommended monitoring (92%) or treatment change (83%); the lowest concordance when the decision was for the “further discussion” option (59%). Patient acceptability was reported by 97% of individuals.

**Conclusions:**

MENTOR is an easy to use tool to monitor the treatment of NBD and determinate progression through the clinical pathway. This validation study shows good correspondence between expert clinician opinion and MENTOR result. The tool has potential to be used in other patient groups, following further studies.

## Introduction

Neurogenic bowel dysfunction (NBD) is a term used to describe gastrointestinal symptoms that complicate lesions or diseases of the central nervous system. The commonest symptoms are constipation and faecal incontinence (FI) [1, 2]. Symptoms of NBD affect the majority of individuals with spinal cord injury (SCI), multiple sclerosis (MS), spina bifida (SB) and cauda equina syndrome with a prevalence of up to 80% depending on the underlying disorder [3–5]. The pathology arises from disturbed neural function affecting both whole gut transit, but also sensory and motor aspects of bowel evacuation [6–8].

Symptoms of NBD have a major impact on quality of life [9, 10] with the loss of independence to achieve or control defecation being a key factor of it [9]. In addition, there is the burden of excessive time spent on toileting and increased frequency of urinary tract infections that accompanies sub-optimal bowel management [11]. For individuals with chronic conditions in the community, the prevalence of NBD may be as high as 98% [12] and 40% report being dissatisfied with their current bowel management [13]. Moreover, a survey of 287 community-dwelling individuals with SCI identified that 70% had not had a change of any aspect of their bowel management in the preceding 5 years [13]. This may have an impact on the fact that hospitalisations are twice as common in people with NBD symptoms compared with those individuals with SCI with well-regulated bowel function [14]. NBD also influences general physical and psychological health [15, 16].

These data are especially dispiriting since there is a well-described stepwise approach to treatment of NBD [17, 18]. The initial intervention usually comprises conservative options such as regulation of diet and fluid intake and use of oral medications (stool softeners and laxatives). This may need to be supplemented by a rectal approach (digital stimulation, suppositories and medicated enemas). If such standard bowel care is insufficient, individuals with SCI may need to escalate therapy to include the minimally invasive treatment option of transanal irrigation (TAI). A minority of this population may consider surgical procedures like the antegrade continence enema or a colostomy [19, 20], or experimental neuromodulation therapies [21]. However, it is clear that management of NBD is not systematically undertaken [13, 17].

Much of the early literature quantifying NBD symptoms used questionnaires developed for patients with idiopathic conditions (Cleveland Constipation score and FI score, or St Mark’s score [22, 23]). A specific NBD score was developed in 2006 and has been translated to several languages and validated across various cultures [24]. It correlates with the impaired quality of life and is sensitive to change. The score was developed for the SCI population and has also been used in MS cohorts [25]. It was not developed for clinical decision-making for individuals presenting with an ineffective bowel regimen [26]. Without a clear definition of treatment failure, it is difficult to understand whether and when it is the right moment to change therapy. Appropriate bowel care prevents complications and hospitalisation, increases quality of life and should be considered a key goal in the management of the chronic SCI population.

This paper describes the creation and validation of Monitoring Efficacy of NBD Treatment On Response (MENTOR) tool. This tool was designed to help healthcare professionals and SCI individuals determine if and when treatment of bowel symptoms needs to be re-assessed from the existing approach.

## Methods

The project comprised two phases; phase I was tool development, and phase II was the validation process.

### Phase I—development of the tool

**Fig. 1:**
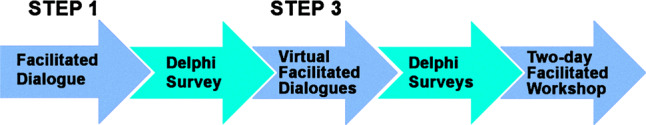
Modified Delphi research methodology.

The methodology used to reach consensus on this project was a Modified Delphi Process that combines the scientific rigour of the traditional Delphi and RAND Nominal Group Technique [27], with virtual and face-to-face professionally facilitated dialogues to allow for the generation of new ideas (Fig. 1). The methodology was designed by an International Association of Facilitators Certified Professional Facilitator^©^, who also had a specialised training in evaluative sciences.

**Step 1:** The process to reach consensus was designed by eight clinical and academic international experts to examine the need for evidence-based clinical guidance for physicians to determine when standard bowel care has failed.

**Step 2:** The expert group defined a survey of 20 questions; this on-line questionnaire was mailed to 76 experts. Thirty-five completed responses were received during the established response time of 4 weeks given with a response rate of 46%.

**Step 3:** A virtual facilitated dialogue among Expert Panel members analysing the survey results, discussing the clinical implications and determining where additional research was needed. Experts agreed about factors to consider in a treatment failure.

**Step 4:** A series of three short Delphi surveys that examined specific aspects of the Step 2 results and filled the gaps identified during the Step 3 analysis and discussions.

**Step 5:** During two-day of Consensus Workshop was reviewed, analysed, tested against the literature and then trasformed into the MENTOR tool.

### MENTOR tool

**Fig. 2:**
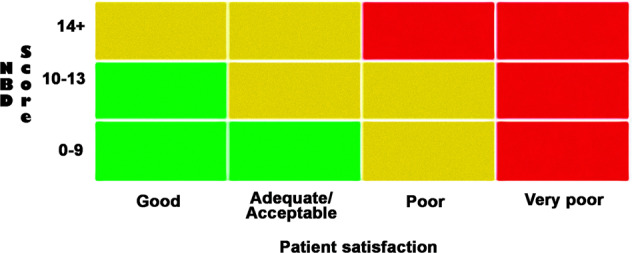
MENTOR grid to allocate participates.

MENTOR is a tool in three dimensions, the components of each are: (1) bowel/defecation symptoms through the NBD score; (2) special attention symptoms (SAS), which are the elements of comorbidity that may be linked to poor bowel management and (3) patient perception of satisfaction with their bowel function (see Appendix 1). Based on each individual’s report of these three factors, it is possible to assign a position in one of three regions of a grid corresponding to a “traffic light” system. These regions are a green area that represents adequate treatment; yellow is sub-optimal treatment that should require discussion with patient which may or may not result in further investigation or treatment change, and consider monitoring after 1–3 months; and red represents inadequate treatment where further investigations and treatment change is likely needed, as shown in Fig. 2.

As mentioned previously, the MENTOR tool is built upon three dimensions; the additional dimension to the NBD score and patient satisfaction being the SAS question. If the participant reports having experienced any of the following symptoms since their last consultation they should immediately be moved one grid square up and one to the right on the grid shown in Fig. 2.

- Intense pain in abdomen or rectum.
- New or increased rectal bleeding.
- Hospitalisation due to bowel problems.
- Loss of independence or change in circumstances that potentially impacts bowel care or bowel function.
- Episode of autonomic dysreflexia related to bowel problems.

Therefore, if a participant were assessed to be in any part of the green zone, any of the conditions or occurrences listed above would move the participant to the yellow zone. If in the yellow zone already, it would mandate that the patient is moved into the red zone. In this way, the presence of any of the SAS creates a mechanism to elevate the “status” of the individual patient to a higher colour level.

### Phase II—validation of the tool

We enroled individuals with SCI from six specialty centres from Europe and the United States, encompassing the setting of four SCI rehabilitation or spinal units as well as two specialist gastroenterology outpatient clinics. The tool was professionally translated from English into the native languages of the participant countries, and then confirmed by certified back translation.

All individuals with SCI had a confirmed diagnosis of non-congenital SCI of more than 3 months of duration, and confirmed NBD with use of at least one method for managing their bowel confirmed by expert clinician and from previous medical records. All were aged over 18 and agreed to participate in the study. Those individuals with impaired ability to read or speak the language of the documentation were excluded. Each participant was identified by a unique, site-specific consecutive code. The MENTOR tool was self-completed in all the three sections prior to the individual with SCI being seen by the expert clinician. A member of the research team registered how long each individual participant took to complete the questionnaire, and verified that the participant had completed all the items. The participants were asked if the tool was easy to understand and complete. The questionnaire was collected before the consultation and the information was not shared with the examining physician. The consultation preceded as per usual practice and the clinician completed the data collection template for physicians (Appendix 2) at the end of the visit. The conclusion of the consultation was one of the following three outcomes: no change (adequate treatment), discuss but no change (after discussion, no change in treatment was made), change (inadequate current treatment, and new or modified management options were proposed).

### Statistical analysis

Data were entered into a pre-determined and locked Excel file. The central data collection coordinator performed the analysis for this validation study; however, the data sent to the central coordinator were blind between centres. The data was analysed using SAS JMP, version 13.1. Statistical significance was accepted as *p* < 0.05. The Shapiro–Wilk/Kolmogorov–Smirnov normality tests analysis were undertaken to assess normality of data distribution. Descriptive statistics was performed. Comparison between groups (e.g. between individuals seen in a rehabilitation setting versus in a gastroenterology outpatient setting) was done through non-parametric tests (Wilcoxon’s two-sample test) when data were not normally distributed.

## Results

We recruited 241 individuals with SCI (age 20–86 years; mean age 49 years (±15 SD), 139 men (58%) and 102 women (42%). Seven participants received more than one consultation during the recruitment period and for this reason they are counted twice for the tool assessment purpose resulting in 248 observations, but just once for demographic statistic purposes.

The severity of SCI was divided into four groups based on the American Spinal Injury Association Impairment Scale classification [28] (AIS Grade E was not included). Causes of non-traumatic SCI represented 47% of our population, included infection, spinal artery occlusion, tethered cord and spinal arteriovenous malformation. In our sample, 61% had an injury level T6 or above, and the majority of injuries were incomplete; 62% (150 of 242) with six missing observations. The UK centre was the largest recruiting site (44% 108 of 248), with the remainder from Netherlands 15% (38 of 248), Italy 12% (30 of 248), USA 11% (26 of 248), Germany 10% (25 of 248) and Denmark 8% (21 of 248). The distribution of our population on the MENTOR grid shows how 42% of participants were classified as red, 33% were green and 25% yellow.

As shown in Table 1, the MENTOR tool and the expert clinician had high overall concordance in their assessment with total agreement of 79%. Concordance was highest for those subjects in the “extremes” of the grid; those classified in green (91%) and red (82%). The agreement rate becomes much lower for those in the intermediate yellow band (59%), where 68% of them in the yellow band did not receive a treatment change recommendation.

**Table 1:** MENTOR results and agreement with healthcare professionals in our sample. MENTOR tool assigned the participant in three different categories: green, yellow or red.

	*N*	%
Sample (*N*):	248	100
Green	81	33
Yellow	63	25
Red	104	42
Concordance green	74	91
Concordance yellow	37	59
Concordance red	85	82
Total concordance	196	79
Yellow + change	20	32
Yellow + no change	43	68
Total treatment change	108	44

Of the 248 completed questionnaires, there was an approximately even split between those seen for a specific problem in a neurogastroenterology clinic (52%) versus those seen for routine review in a rehabilitation clinic/spinal unit setting (48%). The four rehabilitation practices have a combined agreement rate of 62% between the tool and the expert healthcare professionals, whereas in the two specialist neurogastroenterology clinics the combined agreement rate between specialist and tool was 95%. As shown in Table 2, no significant differences were found in the participants coming from the SCI rehabilitation centers versus the gastroenterology clinics for NBD score (*p* > 0.5); the average NBD score for SCI rehabilitation individuals was 11.7 and 13.0 for gastroenterology patients (*p* = 0.6). The average number of SAS was not significantly different either between the two settings; 0.5 in gastroenterology clinics and 0.73 in rehabilitation units (*p* > 0.5).

**Table 2:** Result of MENTOR in gastroenterology versus SCI rehabilitation centres in all three sections of the tool represented in different scale of grey.

	Gastroenterology %	Rehabilitation %	Tot %
NBD < 14	56	65	60
Satisfied	35	27	31
Acceptable	24	45	34
Dissatisfied	25	19	22
Very dissatisfied	16	8	13
No SAS	65	55	60
Any SAS	35	45	40
2 or more SAS	10	18	14

**Fig. 3:**
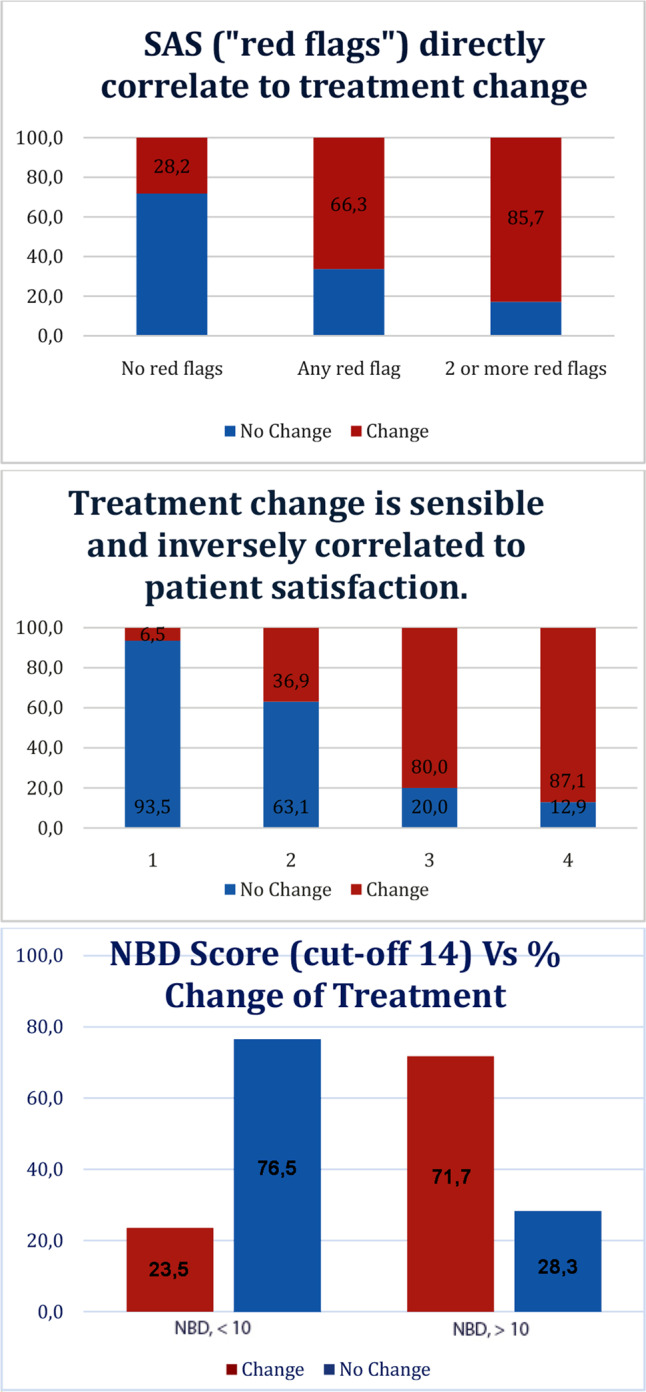
Associations of NBD score, SAS and satisfaction with an expert clinician’s decision of change of treatment.

As show in Fig. 3 there was a linear association between presence of SAS and change of treatment. NBD score value was also associated with the change of treatment. Participant’s satisfaction was inversely correlated with the change of treatment.

The ease of use of the MENTOR tool was assessed based on the objective measure of time taken to complete all three sections, and the subjective perception on easy of completion. The mean completion time was 5.4 min (±2.8 SD) based on 239 observations (nine patients repeated the questionnaire and in the second occasion the time spent to complete was not recorded). We asked the participants to report on the comprehensibility of the tool using a Yes/No question, revealing an approval rate of 97%.

Table 3 shows the data for each centre. The MENTOR tool performed well with similar distribution in the three colour zones within each care contest (SCI rehabilitation, gastroenterology) yet with different distributions between these two settings. However there was better concordance in the gastroenterology than rehabilitation centres, which was most marked in the Yellow zone.

**Table 3:** Results of MENTOR and concordance with healthcare professionals for each center.

%	UK (*n* = 108)	DK (*n* = 21)	TOTAL Gastro (*n* = 129)	DE (*n* = 25)	NL (*n* = 38)	IT (*n* = 30)	US (*n* = 26)	TOTAL Rehab (*n* = 119)	TOTAL (*n* = 248)
Total treat. change	40	86	47	16	26	80	35	39	**44**
Green	42	5	36	28	42	10	35	29	**33**
Yellow	17	33	19	32	40	20	31	31	**25**
Red	41	62	44	40	18	70	34	39	**42**
Total concordance green + red + yellow	94	95	95	44	66	70	66	62	**79**
Concordance green	96	100	97	100	94	0	90	86	**91**
Concordance yellow	95	100	96	0	40	33	50	32	**59**
Concordance red	93	93	93	40	57	91	56	68	**82**

The results in bold are the percentage of our sample not divided in subgroup related to different countries or the two setting.

## Discussion

We present the MENTOR tool, developed and validated by an international team of experts in SCI and NBD. Unanimous agreement was reached among the Expert Panel members, who were confident in their decisions based on the rigour used in the development of the Tool and based on breadth and depth of input from their international colleagues. This tool was designed for persons with SCI, but could also be used for persons with MS and SB.

The tool can be completed by the individual with SCI or carer, and is intended to monitor the efficacy of bowel treatment for individuals with SCI and help clinical decision-making on NBD management.

An initial survey of the literature identified that the evolution of newer therapeutic options for NBD (prokinetic agents, TAI) has not been reflected in change of treatment for participants. This is especially revealing since 40% were dissatisfied with their bowel function [13]. This result has good concordance with our sample where 35% of individuals with SCI were dissatisfied or very dissatisfied with their bowel function. Furthermore, sub-optimal bowel care has a negative impact on quality of life as well as on other physical aspects including urinary tract infections [10, 15]. SCI is a long-term condition, and bowel management strategies are complex and multi-factorial [13, 17]. The case for bowel management being a crucial target in chronic SCI to optimise patient satisfaction and prevent avoidable hospitalisations is clear [4, 14]. The absence of a standard monitoring instrument and reliance on the individual expertise of a clinician increases the variability of bowel care. As such we aimed to create a tool that would be easy to integrate into the standard pathway of care and thus potentially optimise the utilisation of treatment resources.

In subsequent validation of the tool we have obtained evidence that MENTOR performs well when compared with the gold standard of expert clinicians blinded opinion. This was especially true when participants fell in the green and red classifications of the tool. A lower agreement in the yellow area was observed, which may be inevitable in such a hinterland group where the decision on treatment change is more subtle, and where the patient may chose to remain within the safety of a familiar treatment. The concordance was lower in this category because most patients were seen as “no need to change or discuss” by the clinician. In one-third of the Yellow patients an actual change of treatment was considered and enacted. This suggests that patients in the Yellow zone reflect consultations where clinicians are less certain than those with clearly satisfactory or insufficient treatment. It is a strength of the MENTOR tool to help identify those.

There was a notable variation in the prevalence of SCI individuals with unsuccessful bowel treatment, identified in the red area of the grid, between different centres. The services in Italy and Denmark had a high percentage of individuals with SCI in the red area and few in the green one, whilst those in the UK and Netherlands had a higher prevalence of individuals with SCI in green area. Heterogeneity in our sample is most likely explained by differences in the service structure: those in the UK and Netherlands were recruited partially or totally from the cohort returning for an overall SCI problem list review, whilst in Italy and Denmark they were seen in dedicated clinics where individual with SCI were referred for an opinion specifically on their bowel problems. As such, the latter cohort is more likely to score in the red band, and the former group to score in the green. It is noteworthy that despite this variation, there was still good concordance between the tool and clinician (close to 80%), reflecting that MENTOR performs well with people in both these divergent settings. In addition, the very high concordance in the neurogastroenterology units suggests that since the cohort of SCI individuals in those units was not significantly different from those in the SCI units, the MENTOR tool could be used in a rehabilitation setting as a support tool that would provide the rehab professionals with an opinion akin to that of a dedicated GI consultation.

The inclusion of alarm features in the form of SAS is an important component of the MENTOR tool. It reflects the understanding that bowel management has an impact on extraintestinal symptoms and complications. Effective bowel management is known to be associated with reduced gastrointestinal symptoms, autonomic dysreflexia, carer dependence and hospitalisation [7, 9, 11, 14, 29]. As such, the presence of any of these complications is appropriately included in the tool as a feature that should lead to seeking improvements in bowel management. It is notable that each of the three components of the tool correlated to some extent with the gold standard of clinician’s assessment for treatment change. Including all three aspects in one tool improves the sensitivity of the tool without increasing the complexity for the patient completing the questionnaire. This is the first study that we are aware of to evaluate the feasibility and patient acceptability of a modality to assess patient satisfaction with care in NBD. Using the NBD score as single measure of the need to change treatment or not, 72% were offered treatment change. The MENTOR tool combined had a 79% overall agreement and the concordance in Red (when treatment change was performed) was of 82%, so it was somewhat better in both cases than the NBD score alone. Our intention was to create a short tool that combines relevant aspects to measure in this group of individuals with SCI. It is noteworthy that the tool was considered easy to complete by 97% of participants, and that the time taken to complete it was 5 min on average. The symptoms of bowel dysfunction are embarrassing for the patient, and difficult to talk about [30]. Having an instrument which can help reduce the embarrassment of such discussion through writing may help with case finding. The MENTOR tool allows the case finding in a time-efficient way, that is key point to help the clinical pathway.

The MENTOR tool is not intended as a substitute for assessment by a clinician, but it is intended to provide a marker of objectivity to help focus an assessment about the adequacy and to indicate the need to start or change the current bowel management. It may also serve as a useful tool in clinical studies to stratify individuals at entry as well as to demonstrate change after an intervention.

The main limitation of this study is methodological. The tool has been validated against a subjective measure, the decision of an expert clinician. However, in the absence of validated questionnaires for monitoring bowel management in SCI population we considered this a reasonable choice of gold standard. In addition, to avoid bias the MENTOR tool was collected by an independent member of the team and the results of the questionnaire were not revealed to the healthcare professionals who made a clinical decision as per their standard practice.

Another possible limitation is the absence of recording quality of life in our sample. However, the MENTOR tool was designed to reflect adequacy of bowel management, not quality of life, and we felt that inclusion of a quality of life limb would further complicate and lengthen the time taken for the patient to complete the tool. In addition it is important to note that the NBD score has been shown to correlate with quality of life, so this aspect is not completely ignored. Finally, MENTOR tool was translated into a range of languages for the countries where the study was undertaken, and found to be equally comprehensible and usable in all settings.

## Conclusion

MENTOR is a reliable tool to support clinical decision-making for the SCI population with bowel dysfunction. It performed well in routine as well as complication-driven clinical settings. It was similarly acceptable and reflective of clinical practice in a range of countries. The tool provides a holistic assessment of a patient’s symptom burden in a time-effective way. In addition, it may have utility in a rehabilitation setting as a support tool to provide the rehabilitation professionals with an opinion akin to that of a dedicated specialised GI consultation. Further studies would be useful to identify whether it can improve symptoms, reduce hospitalisations, urinary tract infections and other comorbidities in the longer term.

## Supplementary information

Supplemental file

## Data Availability

The datasets generated and analysed during the current study are available from the corresponding author on reasonable request.
