# Supplementary material for: Creation and validation of a new tool for the monitoring efficacy of neurogenic bowel dysfunction treatment on response: the MENTOR tool
Source: Spinal Cord. 2020 Jan 27;58(7):795–802. doi: 10.1038/s41393-020-0424-8 (PMC7340621; doi:10.1038/s41393-020-0424-8)
Supplement: Supplementary file 1 — Supplemental file [file 41393_2020_424_MOESM1_ESM.pdf]

Appendix 1: MENTOR tool

Part 1: The NBD Score. Mark only one answer per question. Your answers should reflect your situation

1. How often do you empty your bowel?
□ Daily (score 0) □ 1-6 times per week (score 1) □ Less than once per week (score 6)

2. How much time do you spend each time to empty your bowel?
□ Less than 30 min. (score 0) □ 31-60 min. (score 3) □More than an hour (score 7)

3. Do you experience uneasiness, sweating or headaches during or after emptying your bowel?
□ Yes (score 2) □ No (score 0)

4. Do you regularly take medication (tablets or capsules) to treat constipation?
□ Yes (score 2) □ No (score 0)

5. Do you regularly take medication (drops or liquid by mouth) to treat constipation?
□ Yes (score 2) □ No (score 0)

6. How often do you use fingers to stimulate and/or empty your bowels (digital stimulation or manual removal)?
□ Less than once per week (score 0) □ Once or more per week (score 6)

7. How often do you experience involuntary loss of stool (bowel accidents)?
□ Daily (score 13) □ 1-6 times a week (score 7) □ 1-4 times a month (score 6) □ A few times a year or less (score 0)

8. Do you take medication to avoid involuntary passing of stool?
□ Yes (score 4) □ No (score 0)

9. Do you experience passing wind (gas or flatus) without being able to control it?
□ Yes (score 2) □ No (score 2)

10. Do you have any skin problems around your anal area?
□ Yes (score 3) □ No (score 0)

Part 2: Have you experienced any of these symptoms since the last time you had a medical consultation regarding your bowel problems?
Tick as many boxes as apply. If none apply, mark "None of the above".

□ Intense pain in your abdomen or rectum
□ New or increased bleeding from the anus
□ Hospitalisation due to bowel problems
□ Reduction in your independence with regard to bowel care
□ An episode of autonomic dysreflexia (pounding headache, profuse sweating…) related to your bowel problems
□ None of the above

Part 3: How would you rate your satisfaction with your bowel functions over the past 4 weeks? Please indicate your overall subjective sensation of satisfaction

□ Good □ Adequate/acceptable □ Poor □ Very poor

Appendix 2: Data collection template for physician

Patient ID:

1. **Injury details:**
   a. Aetiology; traumatic, non-traumatic (if so, of what origin):

   b. Date of injury:
   c. Level:
   d. Completeness:
   e. Hand function: Complete/Partial/No or very limited

2. **ISCOS minimum data set:**
   a. Gastrointestinal or anal sphincter dysfunction unrelated to SCI: Yes / No
   b. Surgical procedures on the gastrointestinal tract: Yes / No

(if Yes, type and date):
   c. Need to wear pad or plug:
   ≥1/day / 1-6 days/week / ≥1/month / ≤1/month / never

3. **Current bowel management (select all that apply):**
- ☐ Specific lifestyle changes (diet, physical exercise, natural supplements including fibers)
- ☐ Non-pharmacological approaches (abdominal massage, biofeedback, pelvic floor training)
- ☐ Digitation (digital stimulation, manual removal of faeces)
- ☐ Bowel medication, oral or rectal (e.g macrogol, laxatives, loperamide, lubiprostone, suppositories, medicated enemas…)
- ☐ Trans-anal irrigation
- ☐ Antegrade irrigation/cecostomy/ACE
- ☐ Nerve stimulation
- ☐ Other surgical approaches/stoma

4. **Independence with bowel care**: Yes / No

5. **Physician's impression of need to change current treatment**: Yes / No
   a. If No go to Q6
   b. If Yes, what is the suggested change (select all that apply)
      i. Need to perform additional investigations
      ii. Change of dose or dosage form (same treatment)
      iii. Change to new treatment (specify which):

Why was this choice made? (Select all that apply)
- ☐ Need for improving symptoms
- ☐ Reduce time spent on bowel management
- ☐ Avoid side effects of current treatment

6. **Final joint decision between clinician and patient**

   Change – Discuss but No Change – No Change
   If answer to Q6 is different to physician's initial choice in Q5, why was this? (free text):

7. **Time to next follow-up**

Legend appendix 1: MENTOR tool
Legend appendix 2: Data collection template for physician completed at the end of each visit
